# Supplementary material for: Effectiveness and Safety of Ayurvedic Medicines in Type 2 Diabetes Mellitus Management: A Systematic Review and Meta-Analysis
Source: Front Pharmacol. 2022 Jun 8;13:821810. doi: 10.3389/fphar.2022.821810 (PMC9213670; doi:10.3389/fphar.2022.821810)
Supplement: Supplementary file 1 [file DataSheet1.zip › Supplementary Material/Forest and Funnel Plots/Sesamum indicum/FBG.pdf]

Sesamum indicum - FBG

Study	Intervention N	Intervention Mean	Intervention SD	Comparator N	Comparator Mean	Comparator SD	Mean Diff. with 95% CI	Weight (%)
Shahi 2017	22	138.59	36.89	22	147.13	54.97	-8.54 [-36.20, 19.12]	48.50
Aslam 2018	23	137.83	15.15	23	218.14	28.39	-80.31 [-93.46, -67.16]	51.50
**Overall**							-45.50 [-115.80, 24.80]	

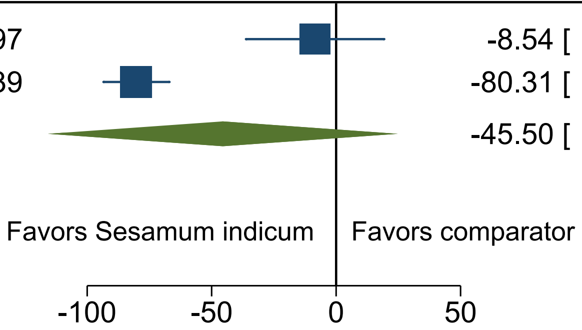

Heterogeneity: $\tau^2 = 2453.35$, $I^2 = 95.26\%$, $H^2 = 21.09$

Test of $\theta_i = \theta_j$: Q(1) = 21.09, p = 0.00

Test of $\theta = 0$: z = -1.27, p = 0.20

Random-effects REML model
